# Primary Cutaneous Melanoma—Management in 2024

**DOI:** 10.3390/jcm13061607

**Published:** 2024-03-11

**Authors:** Anthony Joseph Dixon, Michael Sladden, Christos C. Zouboulis, Catalin M. Popescu, Alexander Nirenberg, Howard K. Steinman, Caterina Longo, Zoe Lee Dixon, Joseph Meirion Thomas

**Affiliations:** 1 Department of Research, Australasian College of Cutaneous Oncology, Docklands, VIC 3008, Australia; alexander.nirenberg@gmail.com (A.N.); zoedixon96@gmail.com (Z.L.D.); 2 Research, American Osteopathic College of Dermatology, Kirksville, MO 63501, USA; 3 Department of Dermatology, University of Tasmania, Launceston, TAS 7005, Australia; m.sladden@doctors.org.au; 4 Departments of Dermatology, Venereology, Allergology and Immunology, Staedtisches Klinikum Dessau, Brandenburg Medical School Theodor Fontane and Faculty of Health Sciences Brandenburg, 06847 Dessau, Germany; christos.zouboulis@mhb-fontane.de; 5 Department of Dermatology, Carol Davila University of Medicine and Pharmacy, 020021 Bucharest, Romania; catalin.m.popescu@gmail.com; 6 Department of Surgery, Campbell University, Buies Creek, NC 27506, USA; hksteinman@gmail.com; 7 Department of Dermatology, University of Modena and Reggio Emilia, 41121 Modena, Italy; caterina.longo@gmail.com; 8 Department of Dermatology, Centro Oncologico ad Alta Tecnologia Diagnostica, Azienda Unità Sanitaria Locale—IRCCS di Reggio Emilia, 41121 Reggio Emilia, Italy; 9 Better Rehab. Occupational Therapy, Surrey Hills, Melbourne, VIC 3127, Australia; 10 Department of Surgery, Formerly of Royal Marsden Hospital, London SW3 6JJ, UK; meirion.thomas@outlook.com

**Keywords:** melanoma, management, treatment, BAUSSS

## Abstract

**Background**: Maximizing survival for patients with primary cutaneous melanomas (melanomas) depends on an early diagnosis and appropriate management. Several new drugs have been shown to improve survival in high-risk melanoma patients. Despite well-documented guidelines, many patients do not receive optimal management, particularly when considering patient age. **Objective**: to provide an update on melanoma management from the time of the decision to biopsy a suspicious skin lesion. **Methods**: We reviewed melanoma-management research published between 2018 and 2023 and identified where such findings impact and update the management of confirmed melanomas. Pubmed, Google Scholar, Ovid and Cochrane Library were used as search tools. **Results**: We identified 81 publications since 2017 that have changed melanoma management; 11 in 2018, 12 in 2019, 10 in 2020, 12 in 2021, 17 in 2022 and 18 in 2023. **Discussion**: Delayed or inaccurate diagnosis is more likely to occur when a partial shave or punch biopsy is used to obtain the histopathology. Wherever feasible, a local excision with a narrow margin should be the biopsy method of choice for a suspected melanoma. The Breslow thickness of the melanoma remains the single most important predictor of outcome, followed by patient age and then ulceration. The BAUSSS biomarker, (Breslow thickness, Age, Ulceration, Subtype, Sex and Site) provides a more accurate method of determining mortality risk than older currently employed approaches, including sentinel lymph node biopsy. Patients with metastatic melanomas and/or nodal disease should be considered for adjuvant drug therapy (ADT). Further, high-risk melanoma patients are increasingly considered for ADT, even without disease spread. Invasive melanomas less than 1 mm thick are usually managed with a radial excision margin of 10 mms of normal skin. If the thickness is 1 to 2 mm, select a radial margin of 10 to 20 mm. When the Breslow thickness is over 2 mm, a 20 mm clinical margin is usually undertaken. In situ melanomas are usually managed with a 5 to 10 mm margin or Mohs margin control surgery. Such wide excisions around a given melanoma is the only surgery that can be regarded as therapeutic and required. Patients who have had one melanoma are at increased risk of another melanoma. Ideal ongoing management includes regular lifelong skin checks. Total body photography should be considered if the patient has many naevi, especially when atypical/dysplastic naevi are identified. Targeted approaches to improve occupational or lifestyle exposure to ultraviolet light are important. Management also needs to include the consideration of vitamin D supplementary therapy.

## 1. Introduction

The two key steps in maximizing survival from primary cutaneous melanomas (melanomas) remain early diagnosis followed by excision with a wide margin. We review the management of melanomas from the time of the decision to obtain a skin lesion biopsy, with a focus on aspects that are sometimes neglected.

We discuss the optimum approach for the biopsy of a suspected melanoma and the subsequent appropriate surgical margins for therapeutic excision.

Several drug interventions have been shown to improve survival in patients with nodal and distant metastases. In the absence of any nodal or wider melanoma spread, adjuvant drug therapy (ADT) is also now considered for high-risk melanoma patients. We review ADT regimens found to improve melanoma survival in randomized controlled trials (RCTs).

We also review the evolving role of sentinel lymph node biopsy (SLNB) in managing melanomas, identify circumstances where it is appropriate and others where SLNB is contraindicated. We summarize the long-term data from RCTs of SLNB.

## 2. Methods

We reviewed melanoma-management research published between 2018 and 2023 and identified where such findings impact and update the management of confirmed melanoma. Pubmed, Google Scholar, Ovid and Cochrane Library were used as search tools.

## 3. Results

We identified 81 publications since 2017 that have changed melanoma management: 11 in 2018, 12 in 2019, 10 in 2020, 12 in 2021, 17 in 2022 and 18 in 2023. Key melanoma strategies that have not been recently impacted recently were reinforced.

Aspects of management that were the subject of updated publications since 2017 included ADT (33 publications), Mohs margin control surgery (10), SLNB (10), surgical margins (7), total body photography (5), mortality risk assessments of melanoma patients (5), occupational therapy considerations after diagnosis (5), skin biopsy techniques (5), as well as histology/pathology, ultrasound and supportive care updates.

## 4. Biopsy Technique

In nearly all cases, the appropriate diagnostic technique for a suspected melanoma is the excision of the entire apparent lesion with a narrow margin, typically 1–3 mm (mm) [1,2,3,4]. The accuracy of an initial diagnosis is significantly better with excisional biopsy (EB) than with partial shave or punch biopsy [2,4,5]. Despite this, shave biopsy remains a common choice, with increasing frequency in some regions [6,7]. An inaccurate assessment of melanoma depth and Subtype may result from partial biopsies, which may increase mortality risk [1]. It was once felt that punch and shave biopsies might increase the risk of melanoma spread [6], but this concept has since been disproven [8].

**Figure 1:**
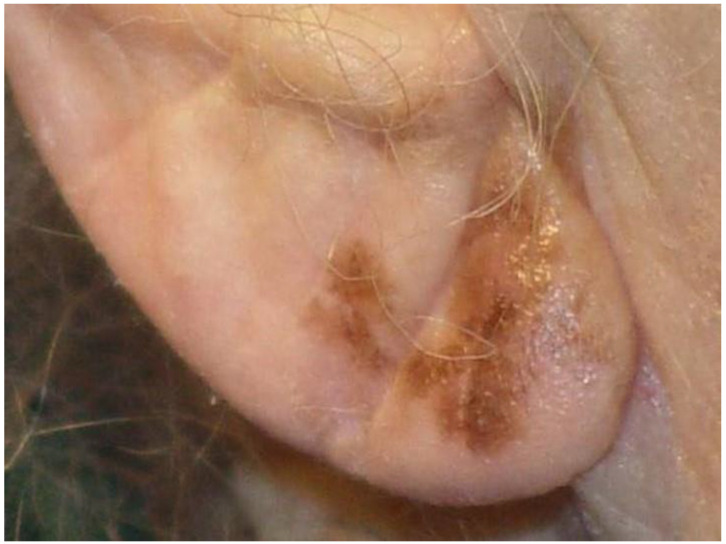
This large pigmented macule on the ear was initially managed with a thick shave biopsy of the entire lesion to confirm diagnosis. The size and location were reasons why local excision was not chosen to biopsy this melanoma.

Partial biopsies are not recommended and should be reserved for circumstances where a complete EB is impracticable [9]. The most common scenario where a partial biopsy is necessary is when the patient has a large lesion on the head, neck, hands, feet, anterior legs or genitalia. Saucerization is an ideal alternative to EB in these circumstances, as long as the specimen includes the entire lesion, including beneath the depth of the lesion throughout. Unfortunately, clinicians who use this technique often do not include the entire melanocytic lesion in their procedure [7,10]. Other options are a deep shave biopsy involving all the pigmented tissue for broad thin tumors [11] or a 6 mm or larger punch biopsy into the adipose layer for thicker tumors [12]. Smaller punch biopsies are especially at risk of incomplete diagnostic assessment and are not recommended [4].

(Figure 1. Large brown macule on right ear was confirmed as a melanoma in situ on a thick shave biopsy).

## 5. Histology Report on Cutaneous Melanoma

An accurate and suitably detailed pathology report is crucial in planning the further management of melanomas. There has been variability in the information relayed in pathology reports; [13,14] hence, recommendations for standardized reports were introduced. These include recommendations initially introduced by the Royal College of Pathologists (RCPath) in 2002 [14,15], which are currently undergoing further revision and update. The RCPath data set includes recommendations for handling skin specimens in the laboratory, and core clinical and pathological data to be included in the report.

**The histology request form:**

Patient age at diagnosis (Age) and sex, the anatomic site of origin (Site) and type of specimen should be provided. Clinical photographs and/or dermatoscopic images of the lesion can be diagnostically helpful by improving clinicopathological correlation.

Macroscopic description information provided should include maximum dimensions of the specimen, including the depth of the specimen, recorded in mms, as well as a description, including dimensions, of any visible lesion.

**The report:**

The microscopic description parameters on the report of a melanoma should include, in relative importance, the following:

a) Breslow thickness/depth (Breslow),

being the depth measured from the top of the epidermis granular layer to the deepest malignant melanocyte;

If the lesion is ulcerated, then Breslow is measured from the base of the ulcer;

The extension of the in situ component along the adnexa and depth of microsatellites are not included in the Breslow;

b) The presence or absence of ulceration (Ulceration), including its diameter in mm;

If the ulcer is traumatic, for example from a previous biopsy, this should be stated on the request form;

c) Subtype of melanoma (Subtype);
d) Margins measured in mm;
e) Growth phase of melanoma;
f) Presence of perineural invasion/neurotropism;
g) Presence of satellites/microsatellites/in-transit metastases;
h) Presence of lymphovascular invasion and extravascular migratory metastases;
i) Tumor-infiltrating lymphocytes,

stated as absent, non-brisk or brisk;

j) Presence or absence of regression;
k) Mitotic index, using the hot spot method;

Although this is no longer used in AJCC staging, its inclusion is recommended.

There are also guidelines for reporting lymph node specimens.

Pathological measurements, both macroscopic and microscopic, are less than clinical measurements, predominantly due to tissue recoil and with a smaller contribution from formalin fixation [16,17].

Pathologist reporting styles vary, including descriptive reports, synoptic reports or a combination of both [13]. However, the recommended data should be included.

## 6. The BAUSSS Biomarker

Until recently, the following nine features from the pathology report and the patient history, in alphabetical order, had been considered important in determining survival prospects: (1) Age, (2) Breslow, (3) Clark level, (4) mitotic activity, (5) Sex, (6) Site, (7) Subtype, (8) tumor regression and (9) Ulceration.

The important study by El Sharouni et al. [18] provides substantial data on patient demographics and pathology characteristics that contribute to survival risk.

These nine features can now be divided into four categories:

Group A. Breslow, Age and Ulceration are the three most important aspects that influence melanoma-specific survival. These are the only aspects with a c-score of over 0.59 [18]

Group B. Subtype, Sex and Site are also important in assessing survival risk but have less impact. These are the only three aspects with a c-score between 0.54 and 0.59 [18]

Group C. Mitotic activity and regression have a minimal impact on outcomes when analyzed on multivariate analysis [18]. The c-statistic of these two features is just over 0.5, indicating their impact on survival prediction is close to random.

Group D. Clark levels describe the tissue layer to which the tumor has descended from the epidermis towards or into the subcutaneous fat. The Clark level provides no statistical difference to outcome on multivariate analysis. Indeed, Clark level is no longer considered a feature of a melanoma worthy of comment in pathology reports [19].

Combining factors from group (A), Breslow, Age and Ulceration with (B) Subtype, Sex, and Site provide the information needed to determine the BAUSSS biomarker method of determining mortality risk [20,21].

The BAUSSS biomarker has emerged as a key staging process in determining melanoma mortality risk [22].

The El Sharouni study identified that the c-score for SLNB is very similar to ulceration [18]. However, SLNB status requires further surgery beyond the information obtained from the pathology report and patient history. The additional information provided by SLNB+BAUSSS compared with BAUSSS alone is minimal, in the order of 3%, with confidence limits often overlapping [22].

## 7. Excision Margins

An invasive melanoma is defined as any melanoma growing into the dermis, having broached the dermo–epidermal junction. Excision margins for wide local excision (WLE) are usually determined using Breslow.

Typical current guidelines recommend the following clinically measured excision margins for melanoma; 10 mm clearance if the Breslow is 1 mm or less, 10 mm to 20 mm clearance if the Breslow is between 1 and 2 mm, and 20 mm if the Breslow is over 2 mm [23]. These radial excision margins are measured clinically with a ruler from the edge of the melanoma [24]. The depth of the excision in usual clinical practice is excision down to but not including the deep fascia. Radial margins greater than 20 mm do not improve survival [25,26,27]. The margins are summarized in Table 1 [24].

### 7.1. Melanoma In Situ Margins

A melanoma in situ (MIS) is a melanoma confined to the epidermis, with no invasive dermal involvement. Traditionally, MIS has been managed with a 5 mm excision margin, but such a clearance can be insufficient if used routinely [28]. The suggestion for margins greater than 5 mm follows concerning recurrence rates when only 5 mm margins are employed [28,29]. Guidelines now generally recommend a margin of 5 to 10 mm with the aim of achieving a complete histological clearance [23,30], while Mohs surgery may be used to obtain clear margins and reduce recurrence for MIS [31,32,33]. A recent systematic review concluded that outcomes with Mohs are comparable to WLE, but considered further evidence was required to confirm a precise role [34]. Mohs has even been studied as an option for invasive melanomas outside of face lesions [31].

### 7.2. Does the Location Change the Margin?

The anatomic location does not alter margin recommendations for melanoma. At times, a particular location might lead to a consideration of Mohs surgery rather than traditional WLE to minimize skin loss. Examples include the eyelid [35], ear [36], lips [37], face [38], hands and feet [39]. In Australia, it has been concerning that many melanoma patients’ tumors are not excised with the required margins of clearance. This mainly occurs when the melanoma location is on the face and is treated with WLE [40,41]. Indeed, only one third of patients in New South Wales and Victoria were treated with appropriate margins based on national guidelines [40,41]. Patients with non-facial tumors were more likely to be overtreated, receiving margins beyond that required for optimum outcomes [41]. There are locations where a 10 mm margin of normal skin may not be possible because of anatomic considerations, such as the eyelid and nasal alar.

### 7.3. Do the Subtype and Site Change the Margin?

The Subtype of a melanoma alters prognosis but does not alter the margins of clearance. Nodular melanoma has a worse prognosis compared with superficial spreading melanoma (SSM) [42,43]. Lentigo maligna (LM) melanomas have a more favorable prognosis than SSM [18]. Acral melanomas, which occur on the palms and soles, have a worse prognosis compared with SSM [18].

Nevertheless, the guidelines around the world for margins of clearance remain the same for all melanomas regardless of Subtype and Site, given there is no evidence that increasing excision margins would improve outcomes [23,44].

## 8. Biopsy and Definitive Excision in a Single Procedure?

In selected European centers, the clinical and dermatoscopic characteristics of the lesion, including a broad estimate of the Breslow being below 1 mm, are used at times to manage MIS and thin melanoma in a single procedure, with 5 mm (MIS) or 10 mm margins (invasive). Further re-excision then occurs only in cases when the estimate was wrong, with the Breslow being >1 mm.

This approach has the advantage of reducing costs by the performing of just one excision and is especially favored when such an excision can be easily performed. There is a lack of prospective data to validate that the required radial margin cannot be obtained in one surgical procedure rather than two.

This approach has the disadvantage that if the clinical diagnosis is incorrect, the patient will have received a sizable defect even though the pathology did not require such. For this reason, the single excision approach is best considered when the clinician is very confident about the clinical/dermoscopic diagnosis.

In the past, WLE was often coupled with SLNB. This was often the reason for encouraging the splitting of EB from the definitive surgery, allowing for both WLE and SLNB at the same time. However, in the BAUSSS era, SLNB has a markedly limited role, with most patients having no indication for SLNB. Further, SLNB can be effectively undertaken at a later stage than the definitive excision when considered valuable for a specific patient [45,46].

### Can One Just Do the Excision Biopsy and Nothing Further?

It has recently been suggested that the days of WLE might be over. Trials to compare the single surgical approach with traditional WLE to using EB alone are envisaged [47]. Such a suggestion hinges on the belief that there is no prospective evidence to support WLE over EB alone.

However, a recent study comparing 126 patients who were managed with biopsy alone compared to 1179 patients managed with WLE demonstrated a mortality hazard ratio (HR) of overall survival of 4.80 (95% CI: 2.05–11.22, *p* < 0.001), and a melanoma-specific survival HR of 2.84 (95% CI: 1.04–7.76, *p* = 0.042 [48]), a survival benefit in favor of WLE.

Whether in one procedure or two, achieving the appropriate margin around melanomas as indicated by the Breslow and current guidelines is required. As such, to now consider an RCT in which one arm has an EB alone would face significant ethical challenges. Some patients in the EB arm of such a trial are likely to die unnecessarily.

## 9. When Is a Sentinel Lymph Node Biopsy (SLNB) Considered?

SLNB became popular in the 1990s. SLNB became a common part of melanoma management because its proponents thought that SLNB, followed by completion lymphadenectomy (CL), if SLNB positive (SLNB+), might save lives. The theory was that by identifying patients who had proven lymph node metastases, only those patients would receive a regional dissection of their nodes.

The two most important long-term prospective RCTs of SLNB are the multicenter selective lymphadenectomy trials, denoted MSLT1 [49,50] and MSLT2 [51]. The MSLT1 randomized patients with a melanoma over 1.2 mm in thickness to SLNB and subsequent CL versus observation alone. MSLT1 demonstrated that SLNB and subsequent CL does not improve overall survival or ten-year melanoma-specific survival [49]. The MSLT2 trial randomized patients who had a positive sentinel node into those having CL versus observation. This showed that even in patients with positive sentinel lymph nodes, CL provides no survival benefit when compared to observation alone [51]. Current evidence suggests that SLNB is no longer a routine requirement in the management of melanomas.

Some clinicians recommend SLNB to determine the likelihood of survival from melanoma, as the prognoses of SLNB+ patients are worse than those of SLNB negative (SLNB-) patients [18]. For this reason, it is recommended that SLNB be discussed with melanoma patients [52]. The HR of mortality for SLNB+ versus SLNB- patients is in the order of 2.4 [49] to 2.7 [18].

However, Breslow and Age are more powerful risk factors for mortality than SLNB+. The landmark El Sharouni study [18] identified the area under the curve “C” statistic (c-stat) for an array of possible mortality risk factors. The c-stat for Breslow is in the order of 0.68 to 0.70. Next, Age has a c-stat in the order of 0.64. Ulceration and SLNB+ are the next most independently significant predictors of mortality, with both SLNB+ and ulceration having c-scores in the order of 0.60 to 0.63.

As previously discussed, the next three most important independent risk factors are Subtype, Sex and Site, all having c-stats in the order of 0.55 to 0.58.

While SLNB status provides prognostic information, it is markedly inferior to BAUSSS. Even when SLNB status is added to BAUSSS, the difference is in the order of only 3%, with confidence limits often overlapping [18,21].

The complication rate associated with SLNB is around 11% [53,54,55]. We have reported our concerns that some melanoma patients are being offered SLNB when there is a proven risk of harm but no apparent survival benefit [56]. Concerns regarding the requirement for patients to undergo SLNB for inclusion in some trials of new drugs has been previously described [30].

## 10. SLNB and Age

Our recent research has found that the benefit of SLNB is a particularly poor prognostic indicator for patients under 40 and over 60 years of age [57].

### 10.1. Young Patients

The specificity of SLNB status is especially problematic in young patients. Consider a 20-year-old female patient with a 0.4 mm Breslow, ulcerated TIb SSM on a lower limb with no mitoses or lymphovascular invasion. Such a patient has a 15 year melanoma-specific mortality prediction (MSMP) of 3%, yet the SLNB-positivity risk is about 12% [58]. Shannon et al. suggest SLNB should be considered because the SLNB risk of being positive is high [59].

If 1000 such 20-year-old patients underwent SLNB, 120 would have a positive result. It is likely these 120 patients would then be offered ADT. It is estimated that only 30 of the 1000 patients would die from their melanoma [60]. The HR specific to this age group demonstrates that there will be two melanoma-specific deaths in this clinical scenario in SLNB- patients for every death in a SLNB+ patient [57]. A total of 1000 patients would receive added surgery and 120 patients would be potentially treated with ADT to hopefully prolong life for 10 patients. The projected adverse events from the surgery and particularly from the ADT would render such a management plan ill-conceived.

### 10.2. Older Patients

For older patients, the problem is the reverse. The sensitivity of SLNB status is poor in patients over 60 years of age. Consider an 80-year-old man with a non-ulcerated 1.8 mm Breslow LM type, Tlla melanoma on the cheek. The SLNB+ prediction rate for such a patient is 2%. Hence, SLNB is unlikely to be offered. Yet, this patient has a long-term MSMP of 27%.

Of 1000 such 80-year-old patients, 270 would be expected to die of their melanoma. It is estimated that only 8 of these 270 deaths (3%) would be SLNB+ patients. The decision to undertake SLNB on such patients along with any decision to initiate ADT based on SLNB results is ill-conceived. Rather, all 1000 patients need consideration for ADT.

### 10.3. Suggested Usage of SLNB in 40- to 60-Year-Old Patients

SLNB can be of benefit by providing important prognostic information in addition to that provided by BAUSSS for patients between 40 and 60 years of age. For those patients considered to be borderline for consideration for ADT, SLNB can refine such mortality risk projections. However, even when this test is offered, it is important that those who are found positive do not then undergo CL.

## 11. Investigations following Diagnosis

### 11.1. Ultrasounds of Nodal Basins

**Figure 2:**
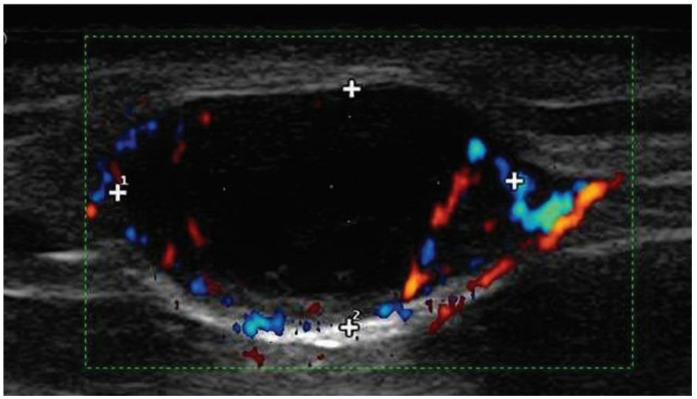
Doppler ultrasound image of a lymph node demonstrating the three key features identified by Dr. Voit in determining survival predictability: (1) loss of central echos, (2) peripheral perfusion and (3) balloon shaping. (Image courtesy of the late Prof. Dr. Christiane Voit).

Voit et al. determined that a non-conventional set of ultrasound (US) criteria more accurately assessed nodal basins in melanoma patients [61,62,63]. A technique called the Berlin Method involves US with the Fine Needle Biopsy (BUSFNB) of nodal beds. The approach developed and trialed in Berlin focuses on three key features: the loss of central echoes, balloon shaping and peripheral perfusion (Figure 2). BUSFNB offers improved sensitivity when compared to the traditional US methodology.

Oude Ophius et al. published long-term data evaluating BUSFNB as a predictor of melanoma-specific survival (MSS) [62]. Their Kaplan Meier graph of MSS patients for SLNB positivity versus negativity are similar to BUSFNB graph of positivity versus negativity [53].

In skilled hands, BUSFNB can identify early melanoma involvement in lymph nodes similarly to SLNB [64]. If US in the best hands could be just as reliable, (hence, just as unreliable) as SLNB status, then its usage must be questioned, especially for patients under 40 and over 60 years of age.

Thompson et al. recently reviewed the use of US in the MSLT2 trial [51,65]. They concluded that preoperative US was not reliable in nodal staging for melanoma patients. The MSLT2 trial used traditional ultrasound (TUS) criteria, assessing hilar vessels, length to depth ratio, the loss of central echoes, and focal areas of low-level echoes surrounded by increased vascularity. Importantly, the BUSFNB factors of balloon shaping and peripheral perfusion were not considered in this study.

Thompson et al. [65] suggested that the BUSFNB method could not be validated in their study group and that patients require SLNB to correctly determine nodal status. However, because their study [51] used TUS and not BUSFNB, their conclusion [65] is invalid. One cannot validate or otherwise an intervention when one does not test the actual intervention.

Ipenburg [66] assessed TUS in melanoma patients who were older or had other health circumstances. In using lymphoscintigraphy and US without fine needle biopsy (FNB), 33% of nodal recurrences were detected before they became clinically apparent. This methodology was considered reasonable for this patient group.

### 11.2. Other Investigations

In 2005, it was understood that no specific investigations, including blood and radiologic tests, were helpful following the diagnosis of melanomas. However, at that time there were no known pharmaceutical approaches shown to prolong survival in melanoma patients. Imaging was often instigated by symptoms and/or signs that might indicate metastatic disease. Now we have options for managing melanoma metastases. The early detection of secondaries may or may not alter the efficacy of newer treatments. The role of early imaging in all asymptomatic patients is of uncertain benefit [67]. However, routine imaging is increasingly considered for high-mortality patients [68]. The BAUSSS biomarker could find a role in determining which asymptomatic-melanoma patients are selected for imaging.

## 12. Adjuvant Drug Therapy

There are now several main groups of drugs commonly in use that have been shown to prolong survival for melanoma patients. These have been reviewed recently and include [69] the following:

**1. Targeted therapy**

B-Raf protein signal (BRAF) inhibitors (dabrafenib, vemurafenib and encorafinib), usually prescribed with mitogen-activated protein kinase (MEK) inhibitors.

MEK inhibitors (trametinib, cobimetinib and binimetinib), usually with BRAF inhibitors.

**2. Immune checkpoint inhibitors**

Inhibitors of the programmed cell death-1 (PD-1) co-receptor (nivolumab, spartalizumab pembrolizumab and toripalimab) or inhibitors of their ligand PD-L1 (atezolizumab).

Inhibitors of the cytotoxic T-lymphocyte-associated protein 4 (CTLA4) receptors (ipilimumab).

Inhibitors of the lymphocyte-activation gene 3 (LAG3) transmembrane protein (relatlimab) or the combination of anti-PD-1 with anti-LAG3 (opdualag).

**3. Emerging drugs and combination therapies**

The medications above which have been shown to provide benefits over years rather than months in melanoma patients with nodal and distant metastatic disease can be prescribed by clinicians familiar with their uses. In USA, Australia and elsewhere, this is often undertaken by a medical oncologist. In parts of Europe, the dermato-oncologist role includes prescribing these melanoma drugs.

### 12.1. When Are Drug Therapies Indicated?

**There are three main categories when ADT is considered for patients with melanoma:**

Nodal spread;

Distant metastases;

High-risk primary melanomas.

#### 12.1.1. Nodal Disease

The AJCC Stage 3 disease refers to patients with nodal diseases. When nodes are clinically involved, surgical resection continues to be the first-line therapy [70,71,72]. ADT may be considered before and/or after surgical resection or when the lymph node disease is not resectable.

#### 12.1.2. Metastatic Melanoma

The AJCC Stage 4 disease refers to patients with distant metastases. Surgery should be offered to patients who have nodal or operable isolated and resectable distant metastatic diseases [73,74]. There is increasing evidence that combining surgery with ADT for such patients improves long-term outcomes [75]. ADT is frequently offered following surgery. If distant metastases are non-resectable, ADT provides the prospect of prolonging survival.

Recent studies involved patients receiving pembrolizumab and surgery when the distant disease was deemed operable [76]. Outcomes are best when patients received pembrolizumab both before and after surgery, than among those who received pembrolizumab only after [77]. Even in this era of ADT, surgery for operable metastatic disease remains an important component in overall management.

#### 12.1.3. High-Risk Primary Cutaneous Melanoma

Recently, a landmark study by Long et al. [78] trialed pembrolizumab on high-risk melanoma patients who had no identifiable metastatic disease and negative SLNB status. This Keynote-716 study was a multicenter, double-blind, placebo-controlled, crossover or rechallenge, randomized, phase-3 trial conducted at 160 academic medical centers and hospitals across 16 countries. The inclusion criteria included patients with a Breslow greater than 2 mm with ulceration and patients with a Breslow over 4 mm regardless of the presence or absence of ulceration. That is, the study included AJCC T3b, T4a and T4b patients. The results demonstrated improved distant-metastasis free survival versus placebo and a continued reduction in the risk of recurrence. Pembrolizumab was FDA-approved for T3b, T4a and T4b patients following this Keynote-716 study. Nivolumab was also approved for the same indications with the same patient groupings following the CHECKMATE76K trial.

Using the BAUSSS biomarker risk tool, T3b, T4a and T4b patients have a long-term MSMR ranging from 6.5% to well over 10 times that rate (65%+). At the lowest risk of the spectrum, a female 20-year-old with a newly diagnosed Breslow 4.1 mm non-ulcerated SSM on the forearm has a long-term MSMR of around 6.5%

Age was not factored into the risk assessment, but patients aged 12 years and older were eligible for enrolment. A deficiency of the study was that patients such as a 70-year-old male with a 3.9 mm non-ulcerated melanoma (lentigo maligna type) on the forehead would not have been eligible for this trial. Yet, such a patient has a 28% long-term MSMR.

In a future trial of this type, patients with perhaps a 10% or 15% or MSMR based on BAUSSS could be considered appropriate to enroll. This would allow for a more accurate assessment of true high-risk patients compared to the Long [78] methodology.

### 12.2. BRAF Inhibitors—Including Vemurafenib, Dabrafenib and Encorafenib

The group of drugs that first demonstrated a survival benefit for melanoma patients is the BRAF inhibitors. BRAF is a human gene that encodes a protein called serine/threonine-protein kinase B-Raf. BRAF is integral to the signaling pathway known as the RAS/MAPK pathway, which controls several important cell functions [79,80]. Inhibiting the BRAF pathway has been shown to prolong survival in melanoma patients with the V600E mutation. Some other variations of the BRAF mutation also respond to BRAF inhibitors [81]. These BRAF V-600 mutations occur in around half of melanoma patients [81].

An initial response rate of approximately 53% has been documented in metastatic melanoma patients, but later the response is abrogated due to the development of resistance mechanisms by the tumor. The association of immunotherapy with targeted therapy can be used to overcome resistance but with no expressive results at a longer follow-up [82]. BRAF therapy is given orally, avoiding hospitalization.

**Vemurafenib**. Vemurafenib was the first BRAF inhibitor shown to benefit melanoma patients [79]. Overall survival at 6 months was 84% with vemurafenib versus 64% with dacarbazine, which acted as the control agent [79]. Just over half of melanoma patients with the BRAF mutation have a response to vemurafenib [81]. Melanoma patients with brain metastases can benefit from vemurafenib therapy [83].

**Dabrafenib**. The second BRAF inhibitor studied extensively is dabrafenib. In a large RCT of metastatic melanoma patients with the BRAF mutation, median progression-free survival with dabrafenib was 5.1 months versus 2.7 months with dacarbazine [80]. This drug also has an established role in patients with brain metastases [84].

**Encorafenib**. This BRAF inhibitor is combined with Binimetinib in the treatment of metastatic melanomas with a BRAF mutation. Among 192 patients, who were treated with this combination with a median follow-up of 16.6 months, the median progression-free survival was 14.9 months [76,77].

The **adverse events** associated with BRAF inhibitors include fatigue, alopecia, photosensitivity, nausea and diarrhea. Arthralgia can be severe and lead patients to ceasing therapy [79,81]. The adverse event profile is similar with dabrafenib und vemurafenib. The most common grade 3–4 adverse events seen in more than 5% of patients in the encorafenib plus binimetinib group were increased γ-glutamyltransferase (9%), increased creatine phosphokinase (7%) and hypertension (6%), while in encorafenib monotherapy, they were group palmoplantar erythrodysaesthesia syndrome (14%), myalgia (10%) and arthralgia (9%). Usage of a BRAF inhibitor without a MEK inhibitor results in around a quarter of patients developing cutaneous squamous cell carcinomas (SCCs) [79,80,83,84] (Table 2).

### 12.3. MEK Inhibitors in Combination with BRAF Inhibitors

Combining MEK inhibitor and BRAF inhibitor treatments was a major step forward because it produced a synergistic therapeutic effect whilst reducing the adverse event profile of BRAF inhibitors alone [85,95]. The mitogen-activated protein kinase (MEK) enzymes work in another step of the RAS/MAPK pathway [85]. MEK inhibitors are delivered orally.

**Trametinib** is commonly administered in conjunction with dabfrafenib. Patients with metastatic melanomas felt better and lived longer on the combination approach [95]. Twelve-month survival was shown to be 72% with combination dabrafenib/trametinib treatment versus vemurafenib alone (65%) [85]. Importantly, the development of new cutaneous squamous carcinomas associated with BRAF inhibitors is almost eliminated with the combined approach [85]. The incidence of alopecia is also lower on the combined therapy, though gastrointestinal adverse events, pyrexia and peripheral oedema increase in incidence [86].

**Binimetinib** is commonly administered in conjunction with encorafenib [96].

**Cobimetinib** is commonly administered in conjunction with vemurafenib [93,97].

It is now standard for melanoma patients to receive combination therapy rather than BRAF therapy. Even patients previously treated with BRAF therapy may benefit when re-challenged with combination therapy [98]. Combined therapy can be continued for many years with patients continuing to receive benefits [86].

While early trials showed benefits in BRAF-positive melanoma patients with distant metastases, recent trials have shown a role of MEK/BRAF therapy in patients that have nodal diseases but no evidence of metastases elsewhere. These Stage 3 patients had a 3-year survival rate of 86% compared to 77% managed with a placebo [88]. Patients in this trial had their involved nodes completely resected prior to drug intervention.

### 12.4. PD-1 Drugs: Pembrolizumab, Nivolumab, Atezolizumab and Toripalimab

Perhaps the most encouraging new melanoma drugs are the programmed cell-death (PD1) drugs. PD-1 is a checkpoint protein on T-cells that inhibits their activity when PD-1 attaches to PD-L1 proteins on some normal and cancer cells [99]. Cancer cells may have large amounts of PD-L1 which inhibits a beneficial immune response. PD-1 drugs inhibit this reaction and boost the immune response [100].

**Pembrolizumab**. Schachter et al. [101] completed an RCT comparing intravenous (IV) pembrolizumab versus IV ipilimumab for melanoma patients with advanced disease. At two years, 55% of the pembrolizumab-treated patients were alive versus 43% for ipilimumab-treated ones. Pembrolizumab has also been found to be effective in melanoma patients with nodal involvement [102]. In total, 15% of patients on pembrolizumab developed grade 3 to 4 adverse events (Table 3). Hypothyroidism (14% of patients) and other endocrine disorders are recognized possible adverse events. A seven-year follow up trial of pembrolizumab versus ipilimumab has recently been published [103]. Seven-year overall survival was 37.8% with pembrolizumab versus 25.3% with ipilimumab.

**Nivolumab**. Weber et al. compared nivolumab to ipilimumab [106] with stage III and IV melanoma patients. Recurrence free survival at twelve months was 70.5% with pembrolizumab versus 60.8% with ipilimumab. Grade 3 or greater adverse events are similar with both PD1 drugs [104,106,107] (Table 3).

**Toripalimab** has a role similar to other PD-1 drugs managing melanomas. However, it has a particular further role in managing mucosal melanomas, including lip and vulva melanoma [108,109,110,111,112].

**Atezolizumab** is a PD-1 drug often combined with other agents. Recent trials have been completed with Cobimetinib and vemurafenib. One trial was specific to intracranial metastatic disease [113]. Another found that the addition of atezolizumab was not beneficial [114].

**irAE** PD1 therapy has recently been shown to produce an array of chronic immune-related adverse events (irAE) that persist even beyond 12 weeks of therapy cessation [115]. Chronic irAEs that were particularly likely to persist included: myocarditis, endocrinopathies, arthritis, xerostomia, neurotoxicities and ocular events. In contrast, irAEs affecting visceral organs (liver, colon, lungs and kidneys) had much lower rates of becoming chronic irAEs.

## 13. Ipilimumab

Ipilimumab is a monoclonal antibody that activates the immune system [99]. It is delivered intravenously. Robert [99] evaluated patients with metastatic melanomas treated with ipilimumab and dacarbazine versus dacarbazine alone. Although dacarbazine has been used for 30 years, its efficacy in managing melanomas is poor [116]. However, it was considered the best agent available for a comparison with newer pharmaceuticals. In total, 20.8% of ipilimumab-plus-dacarbazine patients were alive at 3 years versus 12.2% for dacarbazine alone. Ipilimumab was subsequently found to slow progression in patients with brain metastases [100]. Complications from therapy are concerning, with over half of patients suffering either grade 3 or 4 adverse events (Table 4). Immune-related colitis along with liver impairment are among the concerning sequelae recognized. Other reported complications are diarrhea, dehydration, fatigue, confusion and skin rashes [99,100].

Ipilimumab has been trialed for the indication of post-surgery ADT following nodal resection [75]. Ipilimumab was demonstrated to have an advantage in overall survival as well as distant metastasis-free survival.

In recent years, trials of ipilimumab in combination with other melanoma drugs, especially with nivolumab, have been published. One trial found the combination did not improve recurrence-free survival versus nivolumab alone in patients with nodal and/or metastatic disease. Another long-term trial of the combination [117] showed durable, improved clinical outcomes with the combination over either nivolumab or ipilimumab alone. The adverse event profile with this combination remains concerning (Table 4). A total of 59% of patients developed severe (grade 3) adverse events on combination therapy [104]. Three-year survival was statistically improved, being 58% in the combination therapy group versus 52% on nivolumab alone. There is a particular role of combined therapy for patients with brain metastases [107].

Other studies also suggest a role for patients receiving both pembrolizumab and ipilimumab [76].

There are occasions when the possible improvement in survival from combined therapy that includes ipilimumab may not be in the patient’s best interests considering the adverse events profile. This is a matter always needing considerable discussion with individual patient preferences and needs taken into consideration. Shared decision making is important.

**Table 4:** Ipilimumab with or without Nivolumab.

Drug	Indication	Established MSS * or Recurrence Free Advantage	Grade 3 + Adverse Events
Ipilimumab alone	Unresectable or metastatic melanoma	61% recurrence free at 12 months [106], 34% survival at 3 years [104], 2.9-month median-free survival [105], 43% 2-year survival [101] 65% 5-year recurrence free (Stage III disease) [118]	46% [106], 43% discontinued [106], 2 deaths [106], 28% [104,105], 54% [118]
Ipilimumab and Nivolumab combination	Unresectable or metastatic melanoma	58% survival at 3 years [104] 11.5 month median progression-free survival [105] 75% recurrence-free survival at 12 months [119]	59% [104,105,106,107] 71% [119]

* MSS = melanoma-specific survival.

### Newer Combination Therapies

Recently, an array of other combination drug therapies has emerged for managing metastatic and or nodal involved melanoma. These include Lenvatinib [120], Talimogene [121], Laherparepvec [121], spartalizumab [122,123] and Relatlimab [124,125,126,127], being combined with one or more of the ADT agents outlined above. A review of these emerging options is not a part of this summary. The drug management of advanced melanoma has recently been reported elsewhere [128].

## 14. Total Body Photography (TBP)

TBP as part of the ongoing management of melanoma patients has been controversial. Higher-risk patients stand to benefit from TBP, with second melanomas being found at earlier Breslow as well as a greater percentage of MIS cases [129,130,131]. The role of TBP is unclear in low-risk melanoma patients [129]. As many melanoma patients have multiple other pigmented growths, including dysplastic/atypical nevi, it can be difficult to assess whether a lesion is static or evolving. This has created an established role for TBP at follow-up visits in such patients. A baseline set of images can be undertaken with underwear in place unless there are preexisting naevi requiring documentation beneath underwear. These images then assist the clinician in identifying new and changing lesions at follow-ups. Early second melanomas can be detected with subtle changes in follow-ups when these photographs are available for comparison [132].

## 15. Follow-Up Appointments

Most patients are discharged from follow-up typically five or ten years after diagnosis and rarely even as shortly as one year after diagnosis if there is no evidence of recurrent disease present [133]. Many current melanoma guidelines recommend ceasing the follow-up of patients at 5 years for those with MIS and 10 years for invasive melanoma. Whilst it might be reasonable to cease checking for lymph nodes or palpating the liver after these time periods, patients should not be considered cured and immune from further melanomas at 10 years.

Whilst follow-up appointments frequently involve a check of the original surgical site and regional nodes for evidence of recurrence and metastasis, many do not include a full-skin examination to check for a second melanoma. A full-skin examination is an essential part of routine melanoma follow-up. These checks should occur ideally every six months for five years and at least annually thereafter. It is vital that the members of the treating team agree on who will undertake these tasks, so that there is no presumption that the full check will be performed at the melanoma unit or in the dermatology office or the general practice setting, when in fact no one may be checking the patient’s skin. Patients who have many naevi, especially when dysplastic/atypical naevi are present, should be considered for ongoing skin checks and baseline TBP with clinicians highly skilled in dermatoscopy.

A Californian study showed that 7.9% of melanoma-survivors developed a second melanoma during follow-up [134]. A Dutch study showed a cumulative 5- to 10-year risk of a second melanoma to be 4.6% [135]. This risk did not decline after 10 years. An Australian study has shown that 11.3% of MIS patients developed a second primary cancer, including the risk of further melanomas [136]. Another Australian study demonstrated that this risk of a second melanoma does not diminish with time [137].

We have previously identified the extent to which the prognosis of melanomas is worse as patients are older [57]. This raises the question of how to manage the skin of melanoma patients after their 5 or 10 years of follow-up.

Fifteen years after their first primary melanoma, the patient is 15 years older. Their prognosis of any second melanoma will be affected by this age difference. Yet their increased risk of a second or third melanoma continues. For this reason, the concept of ceasing ongoing skin checks at 5 or 10 years is concerning. Whilst other aspects of managing melanoma could cease at 5 or 10 years, we believe that it is in the melanoma-patient’s interests that regular skin checks continue. The level of evidence to confirm the ideal follow-up period for melanomas is very poor, and so most published guidelines are eminence- rather than evidence-based [138].

## 16. Sun Exposure Advice

As with the management of any patient suffering from sun exposure conditions, melanoma management includes advice on life-long sun protection and UV minimization. Cumulative sun exposure is a risk factor for further melanoma in patients with a history of melanoma [135]. Advice will ideally include the discussion of wearing wide-brimmed or specialized hats, the use of shade, long sleeved clothing and sunglasses. Perhaps the most neglected aspect of reducing UV exposure is to modify the time -of day in which activities are undertaken. A dramatic reduction in potential UV exposure, especially UVB exposure, can be achieved simply by avoiding times when the sun is directly overhead in summer months [139,140].

Patients often require education to understand that current glass technology, especially automobile glass technology, can provide very effective UVB and UVA protection [141]. The Australian population-based Nambour study identified clear melanoma protective benefits in the intervention group who applied sunscreen twice daily compared with the discretionary usage of controls [142].

Sun protection and conscious behavior changes after the diagnosis of melanoma remains critical [143]. Many of our melanoma patients are occupationally exposed to solar UV as part of their work, leisure activities or general activities of daily living. For these patients, occupational therapy (OT) has a role in workplace and home assessments to coordinate safer environments, reducing our patient’s further UV risk [143,144,145,146,147,148,149]. Patients can also benefit from OT through the activity analysis which is undertaken which explores possible adaptations to improve health outcomes. Recent major reviews into more formal systems of reducing ongoing workplace UV exposure have been undertaken [146]. While a given workplace might think they are being effective in minimizing UV exposure to employees, a formal OT worksite assessment can lead to improvements [139,146]. This might include plans to reschedule some outdoor work activities to the early and late parts of the days, and reschedule activities that can be done in the shade or indoors for between 11:00 a.m. and 3:00 p.m.

In the patient’s home, it may be the yard needing a review, such as where are items are stored, whether outdoor kitchen and living areas are protected, and whether outdoor seating arrangements are ideal. Intervention can include recommending a wearable UV-exposure device [150,151,152].

Occupations involving arc welding are at special risk of UV exposure, including to UVC [153]. Delivering education could assist patients and their loved ones to undertake advice and precautions when engaging in occupations where UV exposure is high. Targeted advice can also be geared to recreational UV exposure, such as home gardening [154]. Patients may need to find modifications to continue to enjoy their recreations, but in a safer manner.

## 17. Supplementary Vitamins

Vitamin D oral supplementation needs to be considered for melanoma patients, including measuring blood levels after diagnosis and during follow-up. Apart from providing an alternate source of Vitamin D that is not solar, improved melanoma outcomes may be achievable with such a strategy [155,156,157,158].

The role of nicotinamide supplements is unclear. Nicotinamide has been shown to reduce the development of new squamous cell carcinomas and basal cell carcinomas in skin cancer patients [159,160,161,162]. Its role in kidney-transplant patients is important [163,164]. For melanoma patients, a reduction in new melanoma lesions cannot be expected. However, melanoma outcomes may be improved [165], although further evidence is required. A shorter duration of irAEs associated with PD1 therapy might be achieved with nicotinamide supplementation [166].

## 18. Psychosocial Support

There are many patients for whom the “M” word brings psychological and social difficulties for themselves and their families [167]. Clinicians should consider the effects these important issues have and offer information on psychosocial support services when needed. Psychological support can be provided in the General Practice setting, and melanoma units often have trained staff able to manage these aspects of patient care [167,168,169,170].

## 19. Moving forward—More Trials That Do Not Include SLNB Are Required

Many ADT trials once required SLNB+ to meet the eligibility for enrolment [88,102,106]. This can mean that applicability could be erroneously restricted to those having positive nodes detected by SLNB. This could cement a clinical role for a procedure with no survival benefit and will thus have far-reaching ethical and resource-allocation implications for future patients who may be able to benefit from new interventions. Of particular concern, linking ADT to SLNB will result in ongoing unnecessary drug treatments to young melanoma patients whilst older patients are denied therapies that will prolong their survival.

Patients who decline to have SLNB must not be denied access to drugs that can treat their disease, especially those with a clear high mortality risk on the BAUSSS biomarker.

BRAF/MEK combined therapies are available orally, allowing greater flexibility for melanoma patients who may prefer this to having to attend a chemotherapy center for treatment. PD1 therapy requires parenteral administration.

**Long-term benefit**. Adjuvant drug therapy may provide months or years of additional survival for patients with melanomas. In every RCT of these drugs thus far, the Kaplan–Meier graphic analysis shows a continuing reduction in survival over time. Even though ADT has a proven value, the concept of such being a “cure” for melanoma is not consistent with the data.

**Adverse events**. With all drugs studied, including combination therapies, the severe adverse events demonstrated are concerning both in percentage and nature. Many patients choose to withdraw from the therapies through an intolerance of adverse events. Some patients will choose not to commence these therapies after balancing the potential for extra survival against the risk of side effects.

## 20. Limitations

This review of recent developments in melanoma management is based on the selection of publications since 2017 that the authors considered pertinent. Numerous other melanoma research papers have been written.

## 21. Conclusions

When a cutaneous melanoma is suspected, an excision biopsy should be undertaken with a narrow margin in most circumstances. After diagnosis, a wide excision is required. When clinical/dermatoscopic evidence suggests the melanoma is in situ or invasive but likely <1 mm Breslow thickness, a single diagnostic procedure that also incorporates definitive excision may be appropriate.

The BAUSSS biomarker incorporates the Breslow thickness, the Age of the patient, Ulceration, the Subtype of the tumor, the Sex of patient and the Site of the primary tumor. The BAUSSS provides a more accurate method of assessing high-risk melanoma patients without subjecting patients to the added costs, hospitalization, anesthesia and adverse events associated with SLNB. The BAUSSS is hence integral in the future selection of patients at high mortality risk and hence decisions about adjunctive drug therapy.

Other than surgical and drug management, melanoma patients need regular skin checks to detect possible further primary tumors, with this ideally being lifelong. Management may also require a consideration of vitamin D supplementation. Actions to reduce our patient’s ongoing UV risk can extend beyond the consulting office. Formal worksite and home assessments can identify UV risks and solutions that may not have otherwise been considered. Total body photography has a role in melanoma patients, especially those with many naevi on their skin, especially when these include dysplastic naevi.

## 22. Dedication

**Figure 3:**
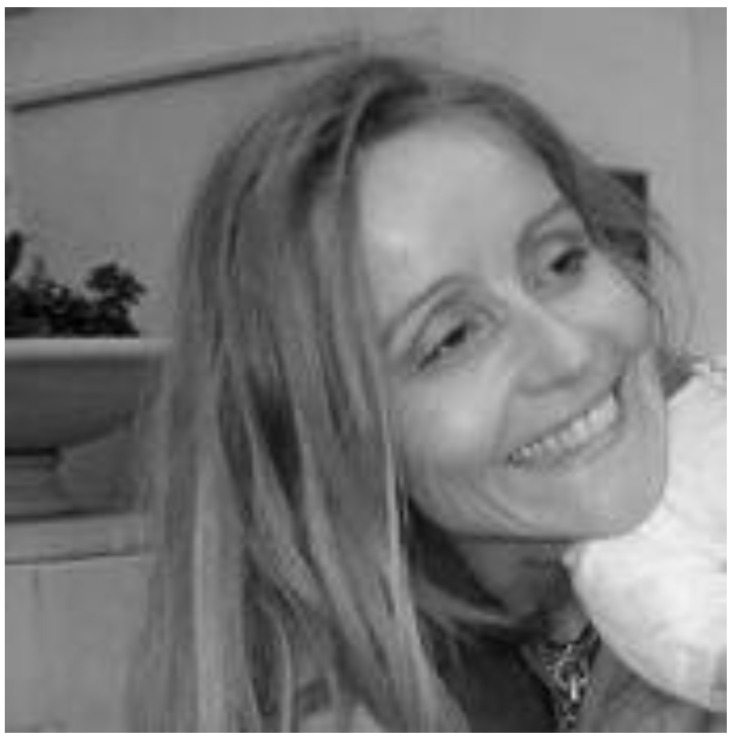
The late Professor Doctor Christiane Voit MD PhD, pioneer of the BUSFNB technique. She is pictured prior to her untimely death from ovarian cancer. (Prof. Dr. Voit requested before her death to publish her photo.)

The authors dedicate this project to the late Dr. Prof. Christiane Voit. Christiane pioneered advancements in melanoma management including the development of an ultrasound approach that improved the accuracy of the detection of nodal disease (Figure 3).

## Figures and Tables

**Table 1:** Recommended margins of excision for primary melanoma are based on Breslow thickness of the tumor.

	Breslow Thickness	Radial Margin of Excision	Depth of Excision
**Melanoma in situ (MIS)**	Not applicable	5 to 10 mm *	Subcutaneous tissue
**Invasive melanoma**	Under 1 mm	10 mm	To but not including deep fascia
	1 to 2 mm	10 to 20 mm **	
	2 to 4 mm	20 mm #	
	Over 4 mm	20 mm ##	

* Some guidelines suggest that MIS margin need only be 5 mm. However, inadequate clearance can be an issue with routine 5 mm margins for MIS. ** Some guidelines suggest that a 10 mm margin is sufficient for all invasive melanoma with Breslow thickness less than 2 mm. # Some guidelines support a 10 mm margin for all invasive melanoma up to 4 mm thick. ## Some guidelines recommend 30 mm margins for thicker tumors [24].

**Table 2:** BRAF inhibitors (Dabrafenib, Vemurafenib or Encorafenib) with or without MEK inhibitors (Trametinib, Cobimetinib or Binimetinib). (Requires tumor to be BRAF V600 mutation-positive).

Drug Combination	Indication in Trial	MSS * Established Benefit	Grade 3 + Adverse Events
Dabrafenib and Trametinib	Unresectable stage 3 or 4 melanoma	72% 12-month survival [85] 44% 3-year survival [86] 28% 5-year survival [87]	41% [86,88]
Dabrafenib and Trametinib	Involvement of lymph nodes following complete resection	58% 3-year relapse-free survival [88] 86% 3-year overall survival [88]	41% [88]
Dabrafenib alone	Unresectable stage 3 or 4 melanoma	32% 3-year survival [86] 5.1 month median progression-free survival [80]	24% [84]
Vemurafenib alone	Unresectable stage 3 or 4 melanoma	65% 12-month survival [85], 17-month median overall survival [89] 15.9-month median overall survival [81] 7.3-month median progression-free survival [90] 17% 4-year survival [91]	61% [92] 28% [89] 59% [93]
Vemurafenib and Cobimetinib	Unresectable or metastatic melanoma	22.3-month median overall survival [89] 9.9-month median progression-free survival [93]	75% [92] 37% [89] 65% [93]
Encorafenib and Binimetinib	Unresectable or metastatic melanoma	14.9 months median progression-free survival [90]	9% increased gammaGT, 6% hypertension, 10% myalgia, 9% arthralgia [90,94]

* MSS = melanoma-specific survival.

**Table 3:** PD1 monotherapy.

Drug	Indication	MSS */Recurrence Advantage	Grade 3 + Adverse Events
Pembrolizumab	Unresectable or metastatic melanoma	55% 2-year survival [101]	32% [101]
Pembrolizumab	Lymph node involvement following complete node dissection	75% 12-month recurrence free [102]	14.7% [102]
Nivolumab	Unresectable or metastatic melanoma	52% three-year survival [104] 6.9-month median progression-free survival [105]	21% [104], 22% [105]
Nivolumab	Lymph node involvement or metastatic disease following complete resection of lymph node involvement or complete resection of metastatic disease	70% 12-month recurrence-free survival [106]	14.4% [106], 10% discontinued [106]

* MSS = melanoma-specific survival.

## Data Availability

Data is contained within the article.

## References

[1] Liszewski W., Stewart J.R., Vidal N.Y., Demer A.M. (2022). Incisional Biopsy Technique Is Associated with Decreased Overall Survival for Cutaneous Melanoma. Dermatol. Surg..

[2] Kok Y., Scott K., Pham A., Liu W., Roberts H., Pan Y., McLean C., Chamberlain A., Kelly J.W., Mar V.J. (2021). The impact of incomplete clinical information and initial biopsy technique on the histopathological diagnosis of cutaneous melanoma. Australas. J. Dermatol..

[3] Restrepo D.J., Huayllani M.T., Boczar D., Sisti A., Gabriel E., Lemini R., Spaulding A.C., Bagaria S., Manrique O.J., Forte A.J. (2019). Biopsy Type Disparities in Patients With Melanoma: Who Receives the Standard of Care?. Anticancer Res..

[4] Ng J.C., Swain S., Dowling J.P., Wolfe R., Simpson P., Kelly J.W. (2010). The impact of partial biopsy on histopathologic diagnosis of cutaneous melanoma: Experience of an Australian tertiary referral service. Arch. Dermatol..

[5] Sharma K.S., Lim P., Brotherston M.T. (2016). Excision versus incision biopsy in the management of malignant melanoma. J. Dermatol. Treat..

[6] Jones S., Henry V., Strong E., Sheriff S.A., Wanat K., Kasprzak J., Clark M., Shukla M., Zenga J., Stadler M. (2023). Clinical Impact and Accuracy of Shave Biopsy for Initial Diagnosis of Cutaneous Melanoma. J. Surg. Res..

[7] Farberg A.S., Rigel D.S. (2016). A comparison of current practice patterns of US dermatologists versus published guidelines for the biopsy, initial management, and follow up of patients with primary cutaneous melanoma. J. Am. Acad. Dermatol..

[8] Martin R.C., Scoggins C.R., Ross M.I., Reintgen D.S., Noyes R.D., Edwards M.J., McMasters K.M. (2005). Is incisional biopsy of melanoma harmful?. Am. J. Surg..

[9] Moscarella E., Pampena R., Palmiotti G., Bonamonte D., Brancaccio G., Piccolo V., Longo C., Argenziano G. (2020). A meta-analysis on the influence of partial biopsy of primary melanoma on disease recurrence and patient survival. J. Eur. Acad. Dermatol. Venereol..

[10] Mir M., Chan C.S., Khan F., Krishnan B., Orengo I., Rosen T. (2013). The rate of melanoma transection with various biopsy techniques and the influence of tumor transection on patient survival. J. Am. Acad. Dermatol..

[11] Saco M., Thigpen J. (2014). A retrospective comparison between preoperative and postoperative Breslow depth in primary cutaneous melanoma: How preoperative shave biopsies affect surgical management. J. Drugs Dermatol..

[12] Pflugfelder A., Weide B., Eigentler T.K., Forschner A., Leiter U., Held L., Meier F., Garbe C. (2010). Incisional biopsy and melanoma prognosis: Facts and controversies. Clin. Dermatol..

[13] Monshizadeh L., Hanikeri M., Beer T.W., Heenan P.J. (2012). A critical review of melanoma pathology reports for patients referred to the Western Australian Melanoma Advisory Service. Pathology.

[14] Taylor L.A., Eguchi M.M., Reisch L.M., Radick A.C., Shucard H., Kerr K.F., Piepkorn M.W., Knezevich S.R., Elder D.E., Barnhill R.L. (2021). Histopathologic synoptic reporting of invasive melanoma: How reliable are the data?. Cancer.

[15] Kaur M.R., Colloby P.S., Martin-Clavijo A., Marsden J.R. (2007). Melanoma histopathology reporting: Are we complying with the National Minimum Dataset?. J. Clin. Pathol..

[16] de Waal J. (2021). Skin tumour specimen shrinkage with excision and formalin fixation-how much and why: A prospective study and discussion of the literature. ANZ J. Surg..

[17] Kerns M.J., Darst M.A., Olsen T.G., Fenster M., Hall P., Grevey S. (2008). Shrinkage of cutaneous specimens: Formalin or other factors involved?. J. Cutan. Pathol..

[18] El Sharouni M.A., Stodell M.D., Ahmed T., Suijkerbuijk K.P.M., Cust A.E., Witkamp A.J., Sigurdsson V., van Diest P.J., Scolyer R.A., Thompson J.F. (2021). Sentinel node biopsy in patients with melanoma improves the accuracy of staging when added to clinicopathological features of the primary tumor. Ann. Oncol..

[19] Owen S.A., Sanders L.L., Edwards L.J., Seigler H.F., Tyler D.S., Grichnik J.M. (2001). Identification of higher risk thin melanomas should be based on Breslow depth not Clark level IV. Cancer.

[20] Dixon A., Steinman H.K., Kyrgidis A., Smith H., Sladden M., Zouboulis C.C., Argenziano G., Apalla Z., Lallas A., Longo C. (2023). Online prediction tools for melanoma survival: A comparison. J. Eur. Acad. Dermatol. Venereol..

[21] Dixon A.J., Steinman H.K., Kyrgidis A., Smith H., Sladden M., Zouboulis C.C., Argenziano G., Apalla Z., Lallas A., Longo C. (2023). Improved methodology in determining melanoma mortality and selecting patients for immunotherapy. J. Eur. Acad. Dermatol. Venereol..

[22] Dixon A.J., Kyrgidis A., Sladden M., Nirenberg A., Steinman H.K., Smith H., Zachary C.B., Anderson S., Leiter-Stöppke U., Longo C. (2024). BAUSSS biomarker further validated as a key risk staging tool for patients with primary melanoma. J. Eur. Acad. Dermatol. Venereol..

[23] Joyce D., Skitzki J.J. (2020). Surgical Management of Primary Cutaneous Melanoma. Surg. Clin. N. Am..

[24] Sladden M.J., Nieweg O.E., Howle J., Coventry B.J., Thompson J.F. (2018). Updated evidence-based clinical practice guidelines for the diagnosis and management of melanoma: Definitive excision margins for primary cutaneous melanoma. Med. J. Aust..

[25] Thomas J.M., Newton-Bishop J., A’Hern R., Coombes G., Timmons M., Evans J., Cook M., Theaker J., Fallowfield M., O’Neill T. (2004). Excision margins in high-risk malignant melanoma. N. Engl. J. Med..

[26] Gillgren P., Drzewiecki K.T., Niin M., Gullestad H.P., Hellborg H., Mansson-Brahme E., Ingvar C., Ringborg U. (2011). 2-cm versus 4-cm surgical excision margins for primary cutaneous melanoma thicker than 2 mm: A randomised, multicentre trial. Lancet.

[27] Sladden M.J. (2012). Sufficiency and safety of 2-cm excision margin for stage IIA through stage IIC cutaneous melanoma. Arch. Dermatol..

[28] Kunishige J.H., Brodland D.G., Zitelli J.A. (2012). Surgical margins for melanoma in situ. J. Am. Acad. Dermatol..

[29] Felton S., Taylor R.S., Srivastava D. (2016). Excision Margins for Melanoma In Situ on the Head and Neck. Dermatol. Surg..

[30] Bigby M., Zagarella S., Sladden M., Popescu C.M. (2019). Time to reconsider the role of sentinel lymph node biopsy in melanoma. J. Am. Acad. Dermatol..

[31] Zitelli J.A., Stiegel E., Brodland D.G. (2023). The Controversy and Value of Mohs Micrographic Surgery for Melanoma and Melanoma in Situ on the Trunk and Extremities. Dermatol. Surg..

[32] Rubin A.I. (2021). Commentary on Mohs Micrographic Surgery Using MART-1 Immunostaining for Nail Unit Melanoma in Situ. Dermatol. Surg..

[33] Phan K., Loya A. (2019). Mohs micrographic surgery versus wide local excision for melanoma in situ: Analysis of a nationwide database. Int. J. Dermatol..

[34] Sharma A.N., Foulad D.P., Doan L., Lee P.K., Atanaskova Mesinkovska N. (2021). Mohs surgery for the treatment of lentigo maligna and lentigo maligna melanoma—A systematic review. J. Dermatolog Treat..

[35] Ramachandran V., Phan K. (2022). Mohs micrographic surgery versus wide local excision for eyelid melanoma: An analysis of a national database. J. Plast. Reconstr. Aesthet. Surg..

[36] Puyana C., Ham P., Tsoukas M.M. (2020). Mohs Micrographic Surgery for the Treatment of External Ear Melanoma: An Outcome Study. Dermatol. Surg..

[37] Queen D., Knackstedt T., Polacco M.A., Collins L.K., Lee K., Samie F.H. (2019). Characteristics of non-melanoma skin cancers of the cutaneous perioral and vermilion lip treated by Mohs micrographic surgery. J. Eur. Acad. Dermatol. Venereol..

[38] Heath M., Woody M., Leitenberger J., Latour E., Bar A. (2020). Invasive Melanoma and Melanoma in Situ Treated with Modified Mohs Micrographic Surgery with En Face Permanent Sectioning: A 10-Year Retrospective Review. Dermatol. Surg..

[39] Isaq N.A., Demer A.M., Vidal N.Y., Lohman M.E. (2023). Dermatologic surgeons’ approaches to acral lentiginous melanoma: A survey of the American College of Mohs Surgery. Arch. Dermatol. Res..

[40] Varey A.H.R., Madronio C.M., Cust A.E., Goumas C., Mann G.J., Armstrong B.K., Scolyer R.A., Curtin A.M., Thompson J.F. (2017). Poor Adherence to National Clinical Management Guidelines: A Population-Based, Cross-Sectional Study of the Surgical Management of Melanoma in New South Wales, Australia. Ann. Surg. Oncol..

[41] Kelly J.W., Henderson M.A., Thursfield V.J., Slavin J., Ainslie J., Giles G.G. (2007). The management of primary cutaneous melanoma in Victoria in 1996 and 2000. Med. J. Aust..

[42] Laeijendecker A.E., El Sharouni M.A., Sigurdsson V., van Diest P.J. (2020). Desmoplastic melanoma: The role of pure and mixed subtype in sentinel lymph node biopsy and survival. Cancer Med..

[43] Lattanzi M., Lee Y., Simpson D., Moran U., Darvishian F., Kim R.H., Hernando E., Polsky D., Hanniford D., Shapiro R. (2019). Primary Melanoma Histologic Subtype: Impact on Survival and Response to Therapy. J. Natl. Cancer Inst..

[44] Fong Z.V., Tanabe K.K. (2014). Comparison of melanoma guidelines in the U.S.A., Canada, Europe, Australia and New Zealand: A critical appraisal and comprehensive review. Br. J. Dermatol..

[45] Leong S.P., Thelmo M.C., Kim R.P., Gokhale R., Rhee J.Y., Achtem T.A., Morita E., Allen R.E., Kashani-Sabet M., Sagebiel R.W. (2003). Delayed harvesting of sentinel lymph nodes after previous wide local excision of extremity melanoma. Ann. Surg. Oncol..

[46] Rodgaard J.C., Kramer S., Stolle L.B. (2014). Sentinel node biopsy (SNB) in malignant melanoma as same day procedure vs delayed procedure: Clinical and economic outcome. J. Plast. Surg. Hand Surg..

[47] Zijlker L.P., Eggermont A.M.M., van Akkooi A.C.J. (2023). The end of wide local excision (WLE) margins for melanoma?. Eur. J. Cancer.

[48] Buja A., Rugge M., Damiani G., De Luca G., Zorzi M., Fusinato R., De Toni C., Vecchiato A., Del Fiore P., Falasco F. (2022). Impact of Wide Local Excision on Melanoma Patient Survival: A Population-Based Study. Front. Public Health.

[49] Morton D.L., Thompson J.F., Cochran A.J., Mozzillo N., Nieweg O.E., Roses D.F., Hoekstra H.J., Karakousis C.P., Puleo C.A., Coventry B.J. (2014). Final trial report of sentinel-node biopsy versus nodal observation in melanoma. N. Engl. J. Med..

[50] Morton D.L., Thompson J.F., Cochran A.J., Mozzillo N., Elashoff R., Essner R., Nieweg O.E., Roses D.F., Hoekstra H.J., Karakousis C.P. (2006). Sentinel-node biopsy or nodal observation in melanoma. N. Engl. J. Med..

[51] Faries M.B., Thompson J.F., Cochran A.J., Andtbacka R.H., Mozzillo N., Zager J.S., Jahkola T., Bowles T.L., Testori A., Beitsch P.D. (2017). Completion Dissection or Observation for Sentinel-Node Metastasis in Melanoma. N. Engl. J. Med..

[52] Sladden M., Zagarella S., Popescu C., Bigby M. (2018). When is a sentinel node biopsy indicated for patients with primary melanoma? Comment on the ‘Australian guidelines for the management of cutaneous melanoma’. Australas. J. Dermatol..

[53] Morton D.L., Cochran A.J., Thompson J.F., Elashoff R., Essner R., Glass E.C., Mozzillo N., Nieweg O.E., Roses D.F., Hoekstra H.J. (2005). Sentinel node biopsy for early-stage melanoma: Accuracy and morbidity in MSLT-I, an international multicenter trial. Ann. Surg..

[54] Espinosa-Pereiro C.E., Zulaica Garate A., Garcia-Doval I. (2019). Complications and Sequelae After Sentinel Lymph Node Biopsy in Melanoma: A Retrospective Cohort Study. Actas Dermosifiliogr..

[55] Moody J.A., Ali R.F., Carbone A.C., Singh S., Hardwicke J.T. (2017). Complications of sentinel lymph node biopsy for melanoma—A systematic review of the literature. Eur. J. Surg. Oncol..

[56] Dixon A., Steinman H., Anderson S., Nirenberg A., Dixon J. (2016). Routine usage of sentinel node biopsy in melanoma management must cease. Br. J. Dermatol..

[57] Dixon A.J., Kyrgidis A., Steinman H.K., Dixon J.B., Sladden M., Garbe C., Lallas A., Zachary C.B., Leiter-Stoppke U., Smith H. (2021). Sentinel lymph node biopsy is unreliable in predicting melanoma mortality for both younger and older patients. J. Eur. Acad. Dermatol. Venereol..

[58] Lo S.N., Ma J., Scolyer R.A., Haydu L.E., Stretch J.R., Saw R.P.M., Nieweg O.E., Shannon K.F., Spillane A.J., Ch’ng S. (2020). Improved Risk Prediction Calculator for Sentinel Node Positivity in Patients With Melanoma: The Melanoma Institute Australia Nomogram. J. Clin. Oncol..

[59] Shannon A.B., Sharon C.E., Straker R.J., Carr M.J., Sinnamon A.J., Bogatch K., Thaler A., Kelly N., Vetto J.T., Fowler G. (2023). Sentinel lymph node biopsy in patients with T1a cutaneous malignant melanoma: A multicenter cohort study. J. Am. Acad. Dermatol..

[60] Zabor E.C., Coit D., Gershenwald J.E., McMasters K.M., Michaelson J.S., Stromberg A.J., Panageas K.S. (2018). Variability in Predictions from Online Tools: A Demonstration Using Internet-Based Melanoma Predictors. Ann. Surg. Oncol..

[61] Ulrich J., van Akkooi A.C., Eggermont A.M., Voit C.A. (2015). Sonographic criteria for diagnosing sentinel node metastases in melanoma patients. Ultraschall Med..

[62] Oude Ophuis C.M.C., Verhoef C., Grunhagen D.J., Siegel P., Schoengen A., Rowert-Huber J., Eggermont A.M.M., Voit C.A., van Akkooi A.C.J. (2017). Long-term results of ultrasound guided fine needle aspiration cytology in conjunction with sentinel node biopsy support step-wise approach in melanoma. Eur. J. Surg. Oncol..

[63] Voit C.A., van Akkooi A.C.J., Catalano O., Eggermont A.M.M. (2017). Pre-SN Ultrasound-FNAC can be Sensitive for Lymph Node Metastases in Melanoma Patients if Performed with the Use of the Berlin Criteria. Ann. Surg. Oncol..

[64] Voit C., Van Akkooi A.C., Schafer-Hesterberg G., Schoengen A., Kowalczyk K., Roewert J.C., Sterry W., Eggermont A.M. (2010). Ultrasound morphology criteria predict metastatic disease of the sentinel nodes in patients with melanoma. J. Clin. Oncol..

[65] Thompson J.F., Haydu L.E., Uren R.F., Andtbacka R.H., Zager J.S., Beitsch P.D., Agnese D.M., Mozzillo N., Testori A., Bowles T.L. (2019). Preoperative Ultrasound Assessment of Regional Lymph Nodes in Melanoma Patients Does not Provide Reliable Nodal Staging: Results from a Large Multicenter Trial. Ann. Surg..

[66] Ipenburg N.A., Thompson J.F., Uren R.F., Chung D., Nieweg O.E. (2019). Focused Ultrasound Surveillance of Lymph Nodes Following Lymphoscintigraphy without Sentinel Node Biopsy: A Useful and Safe Strategy in Elderly or Frail Melanoma Patients. Ann. Surg. Oncol..

[67] Froidevaux S., Calame-Christe M., Schuhmacher J., Tanner H., Saffrich R., Henze M., Eberle A.N. (2004). A gallium-labeled DOTA-alpha-melanocyte- stimulating hormone analog for PET imaging of melanoma metastases. J. Nucl. Med..

[68] Bleicher J., Swords D.S., Mali M.E., McGuire L., Pahlkotter M.K., Asare E.A., Bowles T.L., Hyngstrom J.R. (2020). Recurrence patterns in patients with Stage II melanoma: The evolving role of routine imaging for surveillance. J. Surg. Oncol..

[69] de Oliveira Filho R.S., de Oliveira D.A., Nisimoto M.M., Marti L.C. (2023). A Review of Advanced Cutaneous Melanoma Therapies and Their Mechanisms, from Immunotherapies to Lysine Histone Methyl Transferase Inhibitors. Cancers.

[70] Franke V., van Akkooi A.C.J. (2019). The extent of surgery for stage III melanoma: How much is appropriate?. Lancet Oncol..

[71] Kudchadkar R.R., Michielin O., van Akkooi A.C.J. (2018). Practice-Changing Developments in Stage III Melanoma: Surgery, Adjuvant Targeted Therapy, and Immunotherapy. Am. Soc. Clin. Oncol. Educ. Book.

[72] Bafaloukos D., Gogas H. (2004). The treatment of brain metastases in melanoma patients. Cancer Treat. Rev..

[73] Deutsch G.B., Flaherty D.C., Kirchoff D.D., Bailey M., Vitug S., Foshag L.J., Faries M.B., Bilchik A.J. (2017). Association of Surgical Treatment, Systemic Therapy, and Survival in Patients With Abdominal Visceral Melanoma Metastases, 1965-2014: Relevance of Surgical Cure in the Era of Modern Systemic Therapy. JAMA Surg..

[74] Hau H.M., Tautenhahn H.M., Schoenberg M.B., Atanasov G., Wiltberger G., Morgul M.H., Uhlmann D., Seitz A.T., Simon J.C., Schmelzle M. (2014). Liver resection in multimodal concepts improves survival of metastatic melanoma: A single-centre case-matched control study. Anticancer Res..

[75] Eggermont A.M.M., Chiarion-Sileni V., Grob J.J., Dummer R., Wolchok J.D., Schmidt H., Hamid O., Robert C., Ascierto P.A., Richards J.M. (2019). Adjuvant ipilimumab versus placebo after complete resection of stage III melanoma: Long-term follow-up results of the European Organisation for Research and Treatment of Cancer 18071 double-blind phase 3 randomised trial. Eur. J. Cancer.

[76] Long G.V., Atkinson V., Cebon J.S., Jameson M.B., Fitzharris B.M., McNeil C.M., Hill A.G., Ribas A., Atkins M.B., Thompson J.A. (2017). Standard-dose pembrolizumab in combination with reduced-dose ipilimumab for patients with advanced melanoma (KEYNOTE-029): An open-label, phase 1b trial. Lancet Oncol..

[77] Patel S.P., Othus M., Chen Y., Wright G.P., Yost K.J., Hyngstrom J.R., Hu-Lieskovan S., Lao C.D., Fecher L.A., Truong T.G. (2023). Neoadjuvant-Adjuvant or Adjuvant-Only Pembrolizumab in Advanced Melanoma. N. Engl. J. Med..

[78] Long G.V., Luke J.J., Khattak M.A., de la Cruz Merino L., Del Vecchio M., Rutkowski P., Spagnolo F., Mackiewicz J., Chiarion-Sileni V., Kirkwood J.M. (2022). Pembrolizumab versus placebo as adjuvant therapy in resected stage IIB or IIC melanoma (KEYNOTE-716): Distant metastasis-free survival results of a multicentre, double-blind, randomised, phase 3 trial. Lancet Oncol..

[79] Chapman P.B., Hauschild A., Robert C., Haanen J.B., Ascierto P., Larkin J., Dummer R., Garbe C., Testori A., Maio M. (2011). Improved Survival with Vemurafenib in Melanoma with BRAF V600E Mutation. N. Engl. J. Med..

[80] Hauschild A., Grob J.J., Demidov L.V., Jouary T., Gutzmer R., Millward M., Rutkowski P., Blank C.U., Miller W.H., Kaempgen E. (2012). Dabrafenib in BRAF-mutated metastatic melanoma: A multicentre, open-label, phase 3 randomised controlled trial. Lancet.

[81] Sosman J.A., Kim K.B., Schuchter L., Gonzalez R., Pavlick A.C., Weber J.S., McArthur G.A., Hutson T.E., Moschos S.J., Flaherty K.T. (2012). Survival in BRAF V600-mutant advanced melanoma treated with vemurafenib. N. Engl. J. Med..

[82] Lopes J., Rodrigues C.M.P., Gaspar M.M., Reis C.P. (2022). Melanoma Management: From Epidemiology to Treatment and Latest Advances. Cancers.

[83] McArthur G.A., Maio M., Arance A., Nathan P., Blank C., Avril M.F., Garbe C., Hauschild A., Schadendorf D., Hamid O. (2017). Vemurafenib in metastatic melanoma patients with brain metastases: An open-label, single-arm, phase 2, multicentre study. Ann. Oncol..

[84] Falchook G.S., Long G.V., Kurzrock R., Kim K.B., Arkenau T.H., Brown M.P., Hamid O., Infante J.R., Millward M., Pavlick A.C. (2012). Dabrafenib in patients with melanoma, untreated brain metastases, and other solid tumours: A phase 1 dose-escalation trial. Lancet.

[85] Robert C., Karaszewska B., Schachter J., Rutkowski P., Mackiewicz A., Stroiakovski D., Lichinitser M., Dummer R., Grange F., Mortier L. (2015). Improved overall survival in melanoma with combined dabrafenib and trametinib. N. Engl. J. Med..

[86] Long G.V., Flaherty K.T., Stroyakovskiy D., Gogas H., Levchenko E., de Braud F., Larkin J., Garbe C., Jouary T., Hauschild A. (2017). Dabrafenib plus trametinib versus dabrafenib monotherapy in patients with metastatic BRAF V600E/K-mutant melanoma: Long-term survival and safety analysis of a phase 3 study. Ann. Oncol..

[87] Long G.V., Eroglu Z., Infante J., Patel S., Daud A., Johnson D.B., Gonzalez R., Kefford R., Hamid O., Schuchter L. (2018). Long-Term Outcomes in Patients with BRAF V600-Mutant Metastatic Melanoma Who Received Dabrafenib Combined with Trametinib. J. Clin. Oncol..

[88] Long G.V., Hauschild A., Santinami M., Atkinson V., Mandala M., Chiarion-Sileni V., Larkin J., Nyakas M., Dutriaux C., Haydon A. (2017). Adjuvant Dabrafenib plus Trametinib in Stage III BRAF-Mutated Melanoma. N. Engl. J. Med..

[89] Ascierto P.A., McArthur G.A., Dreno B., Atkinson V., Liszkay G., Di Giacomo A.M., Mandala M., Demidov L., Stroyakovskiy D., Thomas L. (2016). Cobimetinib combined with vemurafenib in advanced BRAF(V600)-mutant melanoma (coBRIM): Updated efficacy results from a randomised, double-blind, phase 3 trial. Lancet Oncol..

[90] Dummer R., Ascierto P.A., Gogas H.J., Arance A., Mandala M., Liszkay G., Garbe C., Schadendorf D., Krajsova I., Gutzmer R. (2018). Encorafenib plus binimetinib versus vemurafenib or encorafenib in patients with BRAF-mutant melanoma (COLUMBUS): A multicentre, open-label, randomised phase 3 trial. Lancet Oncol..

[91] Chapman P.B., Robert C., Larkin J., Haanen J.B., Ribas A., Hogg D., Hamid O., Ascierto P.A., Testori A., Lorigan P.C. (2017). Vemurafenib in patients with BRAFV600 mutation-positive metastatic melanoma: Final overall survival results of the randomized BRIM-3 study. Ann. Oncol..

[92] Dreno B., Ribas A., Larkin J., Ascierto P.A., Hauschild A., Thomas L., Grob J.J., Koralek D.O., Rooney I., Hsu J.J. (2017). Incidence, course, and management of toxicities associated with cobimetinib in combination with vemurafenib in the coBRIM study. Ann. Oncol..

[93] Larkin J., Ascierto P.A., Dreno B., Atkinson V., Liszkay G., Maio M., Mandala M., Demidov L., Stroyakovskiy D., Thomas L. (2014). Combined vemurafenib and cobimetinib in BRAF-mutated melanoma. N. Engl. J. Med..

[94] Dummer R., Ascierto P.A., Gogas H.J., Arance A., Mandala M., Liszkay G., Garbe C., Schadendorf D., Krajsova I., Gutzmer R. (2018). Overall survival in patients with BRAF-mutant melanoma receiving encorafenib plus binimetinib versus vemurafenib or encorafenib (COLUMBUS): A multicentre, open-label, randomised, phase 3 trial. Lancet Oncol..

[95] Grob J.J., Amonkar M.M., Karaszewska B., Schachter J., Dummer R., Mackiewicz A., Stroyakovskiy D., Drucis K., Grange F., Chiarion-Sileni V. (2015). Comparison of dabrafenib and trametinib combination therapy with vemurafenib monotherapy on health-related quality of life in patients with unresectable or metastatic cutaneous BRAF Val600-mutation-positive melanoma (COMBI-v): Results of a phase 3, open-label, randomised trial. Lancet Oncol..

[96] Dummer R., Flaherty K.T., Robert C., Arance A., de Groot J.W.B., Garbe C., Gogas H.J., Gutzmer R., Krajsova I., Liszkay G. (2022). COLUMBUS 5-Year Update: A Randomized, Open-Label, Phase III Trial of Encorafenib Plus Binimetinib Versus Vemurafenib or Encorafenib in Patients with BRAF V600-Mutant Melanoma. J. Clin. Oncol..

[97] Grimaldi A.M., Simeone E., Ascierto P.A. (2015). Vemurafenib plus cobimetinib in the treatment of mutated metastatic melanoma: The CoBRIM trial. Melanoma Manag..

[98] Schreuer M., Jansen Y., Planken S., Chevolet I., Seremet T., Kruse V., Neyns B. (2017). Combination of dabrafenib plus trametinib for BRAF and MEK inhibitor pretreated patients with advanced BRAFV600-mutant melanoma: An open-label, single arm, dual-centre, phase 2 clinical trial. Lancet Oncol..

[99] Robert C., Thomas L., Bondarenko I., O’Day S., Weber J., Garbe C., Lebbe C., Baurain J.F., Testori A., Grob J.J. (2011). Ipilimumab plus Dacarbazine for Previously Untreated Metastatic Melanoma. N. Engl. J. Med..

[100] Margolin K., Ernstoff M.S., Hamid O., Lawrence D., McDermott D., Puzanov I., Wolchok J.D., Clark J.I., Sznol M., Logan T.F. (2012). Ipilimumab in patients with melanoma and brain metastases: An open-label, phase 2 trial. Lancet Oncol..

[101] Schachter J., Ribas A., Long G.V., Arance A., Grob J.J., Mortier L., Daud A., Carlino M.S., McNeil C., Lotem M. (2017). Pembrolizumab versus ipilimumab for advanced melanoma: Final overall survival results of a multicentre, randomised, open-label phase 3 study (KEYNOTE-006). Lancet.

[102] Eggermont A.M.M., Blank C.U., Mandala M., Long G.V., Atkinson V., Dalle S., Haydon A., Lichinitser M., Khattak A., Carlino M.S. (2018). Adjuvant Pembrolizumab versus Placebo in Resected Stage III Melanoma. N. Engl. J. Med..

[103] Robert C., Carlino M.S., McNeil C., Ribas A., Grob J.J., Schachter J., Nyakas M., Kee D., Petrella T.M., Blaustein A. (2023). Seven-Year Follow-Up of the Phase III KEYNOTE-006 Study: Pembrolizumab Versus Ipilimumab in Advanced Melanoma. J. Clin. Oncol..

[104] Wolchok J.D., Chiarion-Sileni V., Gonzalez R., Rutkowski P., Grob J.J., Cowey C.L., Lao C.D., Wagstaff J., Schadendorf D., Ferrucci P.F. (2017). Overall Survival with Combined Nivolumab and Ipilimumab in Advanced Melanoma. N. Engl. J. Med..

[105] Hodi F.S., Chiarion-Sileni V., Gonzalez R., Grob J.J., Rutkowski P., Cowey C.L., Lao C.D., Schadendorf D., Wagstaff J., Dummer R. (2018). Nivolumab plus ipilimumab or nivolumab alone versus ipilimumab alone in advanced melanoma (CheckMate 067): 4-year outcomes of a multicentre, randomised, phase 3 trial. Lancet Oncol..

[106] Weber J., Mandala M., Del Vecchio M., Gogas H.J., Arance A.M., Cowey C.L., Dalle S., Schenker M., Chiarion-Sileni V., Marquez-Rodas I. (2017). Adjuvant Nivolumab versus Ipilimumab in Resected Stage III or IV Melanoma. N. Engl. J. Med..

[107] Tawbi H.A., Forsyth P.A., Algazi A., Hamid O., Hodi F.S., Moschos S.J., Khushalani N.I., Lewis K., Lao C.D., Postow M.A. (2018). Combined Nivolumab and Ipilimumab in Melanoma Metastatic to the Brain. N. Engl. J. Med..

[108] Li S., Wu X., Yan X., Zhou L., Chi Z., Si L., Cui C., Tang B., Mao L., Lian B. (2022). Toripalimab plus axitinib in patients with metastatic mucosal melanoma: 3-year survival update and biomarker analysis. J. Immunother. Cancer.

[109] Li Y.H., Zhou Y., Zhang G.J., Wang Y.W., Wang J.G., Wang X.H., Li Y.F. (2022). Successful treatment of metastatic vulvar malignant melanoma with toripalimab: A rare case report and review of the literature. Medicine.

[110] Lian B., Li Z., Wu N., Li M., Chen X., Zheng H., Gao M., Wang D., Sheng X., Tian H. (2023). Phase II clinical trial of neoadjuvant anti-PD-1 (toripalimab) combined with axitinib in resectable mucosal melanoma. Ann. Oncol..

[111] Lian B., Si L., Chi Z.H., Sheng X.N., Kong Y., Wang X., Tian H., Li K., Mao L.L., Bai X. (2022). Toripalimab (anti-PD-1) versus high-dose interferon-alpha2b as adjuvant therapy in resected mucosal melanoma: A phase II randomized trial. Ann. Oncol..

[112] Tang B., Chi Z., Guo J. (2020). Toripalimab for the treatment of melanoma. Expert. Opin. Biol. Ther..

[113] Dummer R., Queirolo P., Abajo Guijarro A.M., Hu Y., Wang D., de Azevedo S.J., Robert C., Ascierto P.A., Chiarion-Sileni V., Pronzato P. (2022). Atezolizumab, vemurafenib, and cobimetinib in patients with melanoma with CNS metastases (TRICOTEL): A multicentre, open-label, single-arm, phase 2 study. Lancet Oncol..

[114] Ascierto P.A., Stroyakovskiy D., Gogas H., Robert C., Lewis K., Protsenko S., Pereira R.P., Eigentler T., Rutkowski P., Demidov L. (2023). Overall survival with first-line atezolizumab in combination with vemurafenib and cobimetinib in BRAF(V600) mutation-positive advanced melanoma (IMspire150): Second interim analysis of a multicentre, randomised, phase 3 study. Lancet Oncol..

[115] Patrinely J.R., Johnson R., Lawless A.R., Bhave P., Sawyers A., Dimitrova M., Yeoh H.L., Palmeri M., Ye F., Fan R. (2021). Chronic Immune-Related Adverse Events Following Adjuvant Anti-PD-1 Therapy for High-risk Resected Melanoma. JAMA Oncol..

[116] Bajetta E., Di Leo A., Zampino M.G., Sertoli M.R., Comella G., Barduagni M., Giannotti B., Queirolo P., Tribbia G., Bernengo M.G. (1994). Multicenter randomized trial of dacarbazine alone or in combination with two different doses and schedules of interferon alfa-2a in the treatment of advanced melanoma. J. Clin. Oncol..

[117] Wolchok J.D., Chiarion-Sileni V., Gonzalez R., Grob J.J., Rutkowski P., Lao C.D., Cowey C.L., Schadendorf D., Wagstaff J., Dummer R. (2022). Long-Term Outcomes with Nivolumab Plus Ipilimumab or Nivolumab Alone Versus Ipilimumab in Patients with Advanced Melanoma. J. Clin. Oncol..

[118] Eggermont A.M., Chiarion-Sileni V., Grob J.J., Dummer R., Wolchok J.D., Schmidt H., Hamid O., Robert C., Ascierto P.A., Richards J.M. (2016). Prolonged Survival in Stage III Melanoma with Ipilimumab Adjuvant Therapy. N. Engl. J. Med..

[119] Zimmer L., Livingstone E., Hassel J.C., Fluck M., Eigentler T., Loquai C., Haferkamp S., Gutzmer R., Meier F., Mohr P. (2020). Adjuvant nivolumab plus ipilimumab or nivolumab monotherapy versus placebo in patients with resected stage IV melanoma with no evidence of disease (IMMUNED): A randomised, double-blind, placebo-controlled, phase 2 trial. Lancet.

[120] Arance A., de la Cruz-Merino L., Petrella T.M., Jamal R., Ny L., Carneiro A., Berrocal A., Marquez-Rodas I., Spreafico A., Atkinson V. (2023). Phase II LEAP-004 Study of Lenvatinib Plus Pembrolizumab for Melanoma With Confirmed Progression on a Programmed Cell Death Protein-1 or Programmed Death Ligand 1 Inhibitor Given as Monotherapy or in Combination. J. Clin. Oncol..

[121] Chesney J.A., Ribas A., Long G.V., Kirkwood J.M., Dummer R., Puzanov I., Hoeller C., Gajewski T.F., Gutzmer R., Rutkowski P. (2023). Randomized, Double-Blind, Placebo-Controlled, Global Phase III Trial of Talimogene Laherparepvec Combined With Pembrolizumab for Advanced Melanoma. J. Clin. Oncol..

[122] Dummer R., Long G.V., Robert C., Tawbi H.A., Flaherty K.T., Ascierto P.A., Nathan P.D., Rutkowski P., Leonov O., Dutriaux C. (2022). Randomized Phase III Trial Evaluating Spartalizumab Plus Dabrafenib and Trametinib for BRAF V600-Mutant Unresectable or Metastatic Melanoma. J. Clin. Oncol..

[123] Tawbi H.A., Robert C., Brase J.C., Gusenleitner D., Gasal E., Garrett J., Savchenko A., Gorgun G., Flaherty K.T., Ribas A. (2022). Spartalizumab or placebo in combination with dabrafenib and trametinib in patients with BRAF V600-mutant melanoma: Exploratory biomarker analyses from a randomized phase 3 trial (COMBI-i). J. Immunother. Cancer.

[124] Hindie E. (2022). Nivolumab with or without Relatlimab in Untreated Advanced Melanoma. N. Engl. J. Med..

[125] Tawbi H.A., Hodi F.S., Long G.V. (2022). Nivolumab with or without Relatlimab in Untreated Advanced Melanoma. Reply. N. Engl. J. Med..

[126] Tawbi H.A., Schadendorf D., Lipson E.J., Ascierto P.A., Matamala L., Castillo Gutierrez E., Rutkowski P., Gogas H.J., Lao C.D., De Menezes J.J. (2022). Relatlimab and Nivolumab versus Nivolumab in Untreated Advanced Melanoma. N. Engl. J. Med..

[127] Ascierto P.A., Lipson E.J., Dummer R., Larkin J., Long G.V., Sanborn R.E., Chiarion-Sileni V., Dreno B., Dalle S., Schadendorf D. (2023). Nivolumab and Relatlimab in Patients with Advanced Melanoma That Had Progressed on Anti-Programmed Death-1/Programmed Death Ligand 1 Therapy: Results From the Phase I/IIa RELATIVITY-020 Trial. J. Clin. Oncol..

[128] Villani A., Potestio L., Fabbrocini G., Troncone G., Malapelle U., Scalvenzi M. (2022). The Treatment of Advanced Melanoma: Therapeutic Update. Int. J. Mol. Sci..

[129] Ji-Xu A., Dinnes J., Matin R.N. (2021). Total body photography for the diagnosis of cutaneous melanoma in adults: A systematic review and meta-analysis. Br. J. Dermatol..

[130] Hornung A., Steeb T., Wessely A., Brinker T.J., Breakell T., Erdmann M., Berking C., Heppt M.V. (2021). The Value of Total Body Photography for the Early Detection of Melanoma: A Systematic Review. Int. J. Environ. Res. Public Health.

[131] Strunck J.L., Smart T.C., Boucher K.M., Secrest A.M., Grossman D. (2020). Improved melanoma outcomes and survival in patients monitored by total body photography: A natural experiment. J. Dermatol..

[132] Salerni G., Carrera C., Lovatto L., Puig-Butille J.A., Badenas C., Plana E., Puig S., Malvehy J. (2012). Benefits of total body photography and digital dermatoscopy (“two-step method of digital follow-up”) in the early diagnosis of melanoma in patients at high risk for melanoma. J. Am. Acad. Dermatol..

[133] Fernandes N.C., Marinho Fde S. (2015). Evaluation of outpatient discharge in patients with cutaneous melanoma. Rev. Col. Bras. Cir..

[134] Jones M.S., Torisu-Itakura H., Flaherty D.C., Schoellhammer H.F., Lee J., Sim M.S., Faries M.B. (2016). Second Primary Melanoma: Risk Factors, Histopathologic Features, Survival, and Implications for Follow-Up. Am. Surg..

[135] Schuurman M.S., de Waal A.C., Thijs E.J.M., van Rossum M.M., Kiemeney L., Aben K.K.H. (2017). Risk factors for second primary melanoma among Dutch patients with melanoma. Br. J. Dermatol..

[136] Kimlin M.G., Youlden D.R., Brodie A.M., DiSipio T., Youl P., Nair-Shalliker V., Baade P.D. (2018). Risk of second primary cancer in survivors of in situ melanoma. J. Investig. Dermatol..

[137] McCaul K.A., Fritschi L., Baade P., Coory M. (2008). The incidence of second primary invasive melanoma in Queensland, 1982–2003. Cancer Causes Control.

[138] Rueth N.M., Cromwell K.D., Cormier J.N. (2015). Long-term follow-up for melanoma patients: Is there any evidence of a benefit?. Surg. Oncol. Clin. N. Am..

[139] Diffey B.L. (2018). Time and Place as Modifiers of Personal UV Exposure. Int. J. Environ. Res. Public Health.

[140] O’Riordan D.L., Steffen A.D., Lunde K.B., Gies P. (2008). A day at the beach while on tropical vacation: Sun protection practices in a high-risk setting for UV radiation exposure. Arch. Dermatol..

[141] Tuchinda C., Srivannaboon S., Lim H.W. (2006). Photoprotection by window glass, automobile glass, and sunglasses. J. Am. Acad. Dermatol..

[142] Green A.C., Williams G.M., Logan V., Strutton G.M. (2011). Reduced melanoma after regular sunscreen use: Randomized trial follow-up. J. Clin. Oncol..

[143] Idorn L.W., Philipsen P.A., Wulf H.C. (2011). Sun exposure before and after a diagnosis of cutaneous malignant melanoma: Estimated by developments in serum vitamin D, skin pigmentation and interviews. Br. J. Dermatol..

[144] Nasirzadeh N., Monazam Esmaeelpour M., Naseri N., Omari Shekaftik S. (2023). Improving ultraviolet protection properties of cotton textiles using Zinc oxide (ZnO) nanomaterials: An approach for controlling occupational and environmental exposures. Int. J. Environ. Health Res..

[145] Kezic S., van der Molen H.F. (2023). Occupational skin cancer: Measurements of ultraviolet radiation exposure bring knowledge for prevention. Br. J. Dermatol..

[146] Modenese A., Loney T., Rocholl M., Symanzik C., Gobba F., John S.M., Straif K., Silva Paulo M. (2021). Protocol for a Systematic Review on the Effectiveness of Interventions to Reduce Exposure to Occupational Solar UltraViolet Radiation (UVR) Among Outdoor Workers. Front. Public Health.

[147] Beck N., Balanay J.A.G., Johnson T. (2018). Assessment of occupational exposure to heat stress and solar ultraviolet radiation among groundskeepers in an eastern North Carolina university setting. J. Occup. Environ. Hyg..

[148] Nakashima H., Utsunomiya A., Takahashi J., Fujii N., Okuno T. (2016). Hazard of ultraviolet radiation emitted in gas metal arc welding of mild steel. J. Occup. Health.

[149] Downs N.J., Harrison S.L., Chavez D.R., Parisi A.V. (2016). Solar ultraviolet and the occupational radiant exposure of Queensland school teachers: A comparative study between teaching classifications and behavior patterns. J. Photochem. Photobiol. B.

[150] Robinson J.K., Durst D.A., Gray E., Kwasny M., Heo S.Y., Banks A., Rogers J.A. (2021). Sun exposure reduction by melanoma survivors with wearable sensor providing real-time UV exposure and daily text messages with structured goal setting. Arch. Dermatol. Res..

[151] Thieden E., Philipsen P.A., Sandby-Moller J., Wulf H.C. (2005). Sunburn related to UV radiation exposure, age, sex, occupation, and sun bed use based on time-stamped personal dosimetry and sun behavior diaries. Arch. Dermatol..

[152] Thieden E., Philipsen P.A., Heydenreich J., Wulf H.C. (2004). UV radiation exposure related to age, sex, occupation, and sun behavior based on time-stamped personal dosimeter readings. Arch. Dermatol..

[153] Dixon A.J., Dixon B.F. (2004). Ultraviolet radiation from welding and possible risk of skin and ocular malignancy. Med. J. Aust..

[154] Idorn L.W., Thieden E., Philipsen P.A., Wulf H.C. (2013). Influence of having a home garden on personal UVR exposure behavior and risk of cutaneous malignant melanoma in Denmark. Int. J. Cancer.

[155] Bolerazska B., Durovcova E., Marekova M. (2017). Potential of Using Vitamin D as an Adjuvant Treatment of Malignant Melanoma. Klin. Onkol..

[156] De Smedt J., Van Kelst S., Boecxstaens V., Stas M., Bogaerts K., Vanderschueren D., Aura C., Vandenberghe K., Lambrechts D., Wolter P. (2017). Vitamin D supplementation in cutaneous malignant melanoma outcome (ViDMe): A randomized controlled trial. BMC Cancer.

[157] Johansson H., Spadola G., Tosti G., Mandala M., Minisini A.M., Queirolo P., Aristarco V., Baldini F., Cocorocchio E., Albertazzi E. (2021). Vitamin D Supplementation and Disease-Free Survival in Stage II Melanoma: A Randomized Placebo Controlled Trial. Nutrients.

[158] Moreno-Arrones O.M., Zegeer J., Gerbo M., Manrique-Silva E., Requena C., Traves V., Nagore E. (2019). Decreased vitamin D serum levels at melanoma diagnosis are associated with tumor ulceration and high tumor mitotic rate. Melanoma Res..

[159] Ballotti R., Healy E., Bertolotto C. (2019). Nicotinamide as a chemopreventive therapy of skin cancers. Too much of good thing?. Pigment Cell Melanoma Res..

[160] Damian D.L. (2017). Nicotinamide for skin cancer chemoprevention. Australas. J. Dermatol..

[161] Chen A.C., Martin A.J., Dalziell R.A., McKenzie C.A., Lowe P.M., Eris J.M., Scolyer R.A., Dhillon H.M., Vardy J.L., Bielski V.A. (2016). A phase II randomized controlled trial of nicotinamide for skin cancer chemoprevention in renal transplant recipients. Br. J. Dermatol..

[162] Chen A.C., Martin A.J., Choy B., Fernandez-Penas P., Dalziell R.A., McKenzie C.A., Scolyer R.A., Dhillon H.M., Vardy J.L., Kricker A. (2015). A Phase 3 Randomized Trial of Nicotinamide for Skin-Cancer Chemoprevention. N. Engl. J. Med..

[163] Yelamos O., Halpern A.C., Weinstock M.A. (2017). Reply to ‘A phase II randomized controlled trial of nicotinamide for skin cancer chemoprevention in renal transplant recipients’. Br. J. Dermatol..

[164] Drago F., Ciccarese G., Cogorno L., Calvi C., Marsano L.A., Parodi A. (2017). Prevention of non-melanoma skin cancers with nicotinamide in transplant recipients: A case-control study. Eur. J. Dermatol..

[165] Scatozza F., Moschella F., D’Arcangelo D., Rossi S., Tabolacci C., Giampietri C., Proietti E., Facchiano F., Facchiano A. (2020). Nicotinamide inhibits melanoma in vitro and in vivo. J. Exp. Clin. Cancer Res..

[166] De Giorgi V., Colombo J., Trane L., Silvestri F., Venturi F., Zuccaro B., Doni L., Stanganelli I., Covarelli P. (2022). Cutaneous immune-related adverse events and photodamaged skin in patients with metastatic melanoma: Could nicotinamide be useful?. Clin. Exp. Dermatol..

[167] Fischbeck S., Imruck B.H., Blettner M., Weyer V., Binder H., Zeissig S.R., Emrich K., Friedrich-Mai P., Beutel M.E. (2015). Psychosocial Care Needs of Melanoma Survivors: Are They Being Met?. PLoS ONE.

[168] Oliveria S.A., Shuk E., Hay J.L., Heneghan M., Goulart J.M., Panageas K., Geller A.C., Halpern A.C. (2013). Melanoma survivors: Health behaviors, surveillance, psychosocial factors, and family concerns. Psychooncology.

[169] Rychetnik L., McCaffery K., Morton R., Irwig L. (2013). Psychosocial aspects of post-treatment follow-up for stage I/II melanoma: A systematic review of the literature. Psychooncology.

[170] Tan J.D., Butow P.N., Boyle F.M., Saw R.P., O’Reilly A.J. (2014). A qualitative assessment of psychosocial impact, coping and adjustment in high-risk melanoma patients and caregivers. Melanoma Res..

